# Excited States Computation of Models of Phenylalanine Protein Chains: TD-DFT and Composite CC2/TD-DFT Protocols

**DOI:** 10.3390/ijms23020621

**Published:** 2022-01-06

**Authors:** Marine Lebel, Thibaut Very, Eric Gloaguen, Benjamin Tardivel, Michel Mons, Valérie Brenner

**Affiliations:** LIDYL, CEA, CNRS, Université Paris-Saclay, 91191 Gif-sur-Yvette, France; laiguillon32@hotmail.fr (M.L.); thibaut.very@idris.fr (T.V.); eric.gloaguen@cea.fr (E.G.); benjamin.tardivel@cea.fr (B.T.); michel.mons@cea.fr (M.M.)

**Keywords:** electronic excitations, protein modeling, molecular simulation, time-dependent density functional theory, photophysics

## Abstract

The present benchmark calculations testify to the validity of time-dependent density functional theory (TD-DFT) when exploring the low-lying excited states potential energy surfaces of models of phenylalanine protein chains. Among three functionals suitable for systems exhibiting charge-transfer excited states, LC-ωPBE, CAM-B3LYP, and ωB97X-D, which were tested on a reference peptide system, we selected the ωB97X-D functional, which gave the best results compared to the approximate coupled-cluster singles and doubles (CC2) method. A quantitative agreement for both the geometrical parameters and the vibrational frequencies was obtained for the lowest singlet excited state (a ππ* state) of the series of capped peptides. In contrast, only a qualitative agreement was met for the corresponding adiabatic zero-point vibrational energy (ZPVE)-corrected excitation energies. Two composite protocols combining CC2 and DFT/TD-DFT methods were then developed to improve these calculations. Both protocols substantially reduced the error compared to CC2 and experiment, and the best of both even led to results of CC2 quality at a lower cost, thus providing a reliable alternative to this method for very large systems.

## 1. Introduction

Understanding the electronic dynamics of bio-relevant systems usually requires the exploration of potential energy surfaces (PES) of their low-lying excited states, in particular in order to localize non-equilibrium geometries such as conical intersections (CI) [1,2,3,4,5]. This can be accomplished by performing non-adiabatic dynamic simulations considering all the low-lying significant excited states for the dynamics of these systems. A good compromise between accuracy and computational times in simulations on medium-sized systems is to use a single reference method for electronic structure calculations, providing that the excited states are obviously dominated by single excitations. Among the single reference methods, the time-dependent density functional theory (TD-DFT) [6], which describes excited states within response theory, exhibits a favorable cost–performance ratio, but even to only perform a qualitative exploration of the PES, the functional has to be judiciously chosen according to the nature of the excited states considered [7,8,9,10,11]. In this context, we developed an original, innovative computational strategy in order to document the conformer selective dynamics of capped peptide building blocks (including the phenylalanine (Phe) residue), serving as models of proteins [12,13,14,15,16,17,18,19]. Gas-phase isolation was investigated first, enabling cross-checking between the experimental data and relevant quantum chemistry methods in order to validate the theoretical approach. The challenge in such calculations is that these systems exhibit specificities that orient the choice of the approach: (i) their size (medium-size systems where the smallest one, a capped peptide with one residue, already contains at least thirty atoms); (ii) their lack of symmetry, (iii) their great flexibility due to the non-covalent interactions that govern their structure; and (iv) their multiple singlet close-low-lying excited states featuring very different natures, locally excited states on one or several peptide bonds (nπ*_CO_) or on one or several phenyl rings (ππ*) and even charge transfer (CT) states from the backbone to a phenyl ring (nπ*) [17,18,19,20]. This justifies the “multiscale” character of our strategy, which consists of, first, non-adiabatic dynamics simulations within the TD-DFT framework to provide hints about the critical motions that drive the deactivation and then refine them at two better levels of theory. The two better levels of theory are (i) the standard approximate coupled-cluster singles and doubles method (CC2) [21,22,23,24,25] and (ii) a multireference configuration interaction (MRCI) method [26,27].

The first key point in the development of this strategy was to demonstrate, by comparison with multireference configuration interaction (MRCI) calculations on such systems, the validity of the CC2 method. The investigated geometries corresponded not only to the equilibrium geometry of the initially excited state accessible from the Franck–Condon region but also to the equilibrium geometries of all close-low-lying excited singlet states of these systems. In addition, relevant geometries along with the energy profile of the deactivation mechanisms, such as the CI between excited states [17,19], were also considered.

In the second key step, an extensive benchmark of the CC2 method (structure, energetic and vibrational frequencies of the first ππ* excited state) was performed on a series of capped peptides of increasing size and containing several residues, by comparison with experimental data in order to assess the accuracy of this method [18].

Finally, the third key point that remained to be solved consisted in assessing the ability of the TD-DFT method to model the PES of these systems qualitatively. This is one of the objectives of the present work. We first focused on the choice of the functional to be used for performing the calculations (including energetics as well as the first and second derivatives of the energy) on a series of capped peptides of increasing size and containing several different residues. These were chosen for their ability to adopt in the ground state prototypical secondary structures of proteins [15]. Due to the presence of CT states in these systems, benchmarking calculations were performed using three long-range corrected hybrid functionals, LC-ωPBE [28], CAM-B3LYP [29], and ωB97X-D [30] that are suited for the calculations of such states [8,9,31]. These calculated data were directly compared with both experimental spectroscopic data and results of the more refined, correlated, single reference CC2 method [18]. The second objective of this work, moreover, was to evaluate composite CC2/TD-DFT protocols for the 0–0 excitation energies calculations and to determine if such protocols can become, for large and very large-sized systems, an alternative to more refined single reference methods such as CC2, which become not feasible in this case. Indeed, it has recently been demonstrated on several series of organic and inorganic molecules that, while the vertical excitation energies are sensitive to the choice of the method, CC2 or TD-DFT, the difference in zero-point vibrational energy (ZPVE) corrections between the excited and ground states as well as the excited state relaxation energy is much less sensitive to it [32,33,34,35]. Therefore, it would be desirable to develop and assess such composite protocols capable of yielding 0–0 excitation energies of CC2 quality at a lower cost. In this regard, we investigated two types of protocols: the first one that does not use CC2 for both the geometry optimizations and frequency calculations and the second one that uses it only for the geometry optimizations.

## 2. Results and Discussion

### 2.1. Selection of the Functional in TD-DFT Calculations

**Figure 1:**
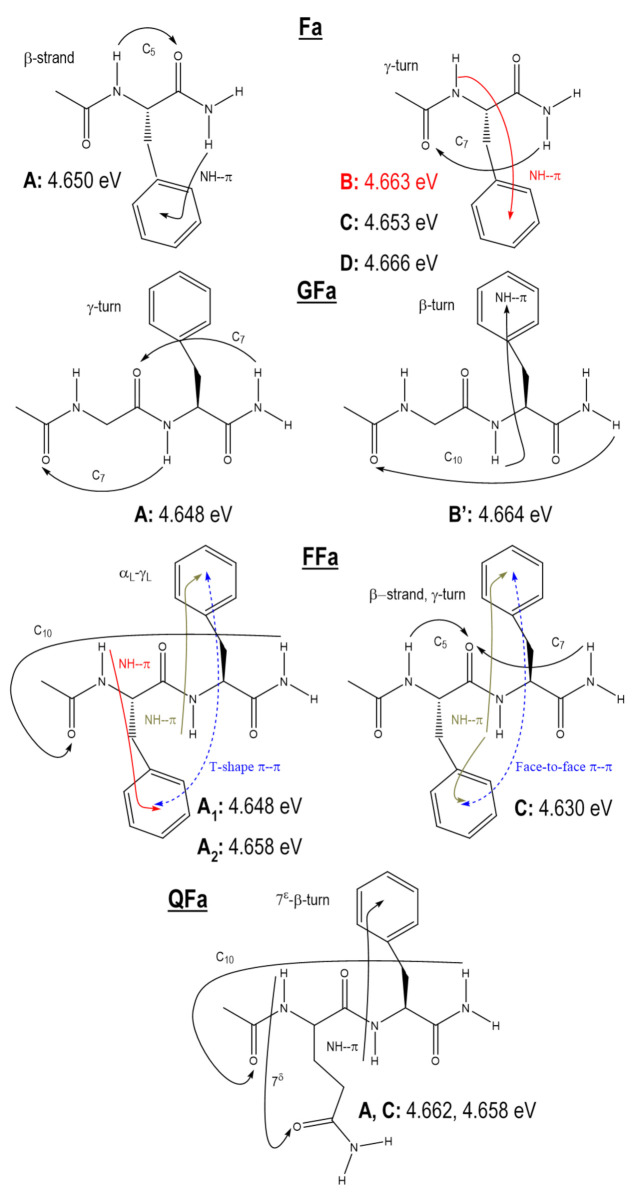
Ac-Phe-NH_2_ (Fa) and Ac-X-Phe-NH_2_ with X = Gly, Phe, Gln (GFa, FFa, and QFa, respectively) conformers and their experimental 0–0 transition energies (eV). Arrows indicate non-covalent intramolecular bonds (hydrogen bonds (C_5_, C_7_, and C_10_), NH-π bonds, and phenyl ring–ring arrangements). Letters A, B, … refer to the notation adopted in the experimental reports to designate the conformers observed, which correspond to different types of backbone folding and non-covalent interactions (see the Methods section for a detailed description). In the case of several S_0_ conformers, which present both similar backbone folding and non-covalent networks, a single quotation mark is put to distinguish them, such as B and B’. In the case of several S_1_ conformers whose conformations have been obtained from the geometry optimization of the same S_0_ conformer but correspond to different excitation such as excitation via each chromophore, a number is put in subscript to distinguish them, such as A_1_ and A_2_.

The selection of the functional was carried out in three steps on the reference system, the *N*-acetyl-phenylalaninyl-amide (Ac-Phe-NH_2_, Fa in short), and more specifically on its four conformers (Fa A-D) identified in the experiment (Figure 1) and characterized by CC2 calculations [18,36]. First, at the DFT-D optimized geometry (B97-D2/TZVPP) of the ground state, the TD-DFT/cc-pVDZ five lowest excited states of the four conformers and their vertical excitation energies were analyzed by comparison with those obtained at the CC2/cc-pVDZ level. Second, the geometry optimization of the lowest excited state accessible from the Franck–Condon region, a singlet ππ* excited state localized on the phenyl ring, was performed at the TD-DFT/cc-pVDZ level for each of the four conformers and the quality of the optimized geometries obtained was assessed by comparison with the CC2 ones. Finally, for each of the four conformers, the adiabatic ZPVE-corrected excitation energies and the IR signature, the *amide A* vibrational frequencies (NH stretches), of this lowest ππ* excited state were evaluated at the TD-DFT/cc-pVDZ level and compared to CC2/cc-pVDZ results as well as to experimental data.

#### 2.1.1. Nature and Vertical Excitation Energies for the Five Lowest Excited States of Fa Conformers

**Figure 2:**
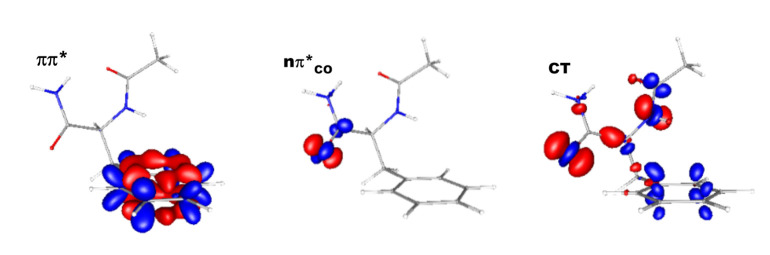
Contours (ππ* ± 0.0015 au, nπ*_CO(2)_ ± 0.03 au, and CT ± 0.007 au) of the CC2 electron density difference between excited and ground states, calculated at the B97-D2/TZPP optimized geometry of the Fa B ground state. A density increase (decrease) is indicated in blue (red).

In the ground state, four conformers (A–D) with two different types of backbone folding were observed and assigned to conformations lying in an energy range of 0–6.5 kJ/mol (DFT-D level, Figure S1a,b, and Table S1). At the CC2 level, whatever the conformers, the first and the fourth excited states were locally ππ* excited states centered on the phenyl ring (see Table 1, Table S2a–c, and Figure 2). The second and the third were locally nπ*_CO_ excited states, each one centered on a peptide bond; the first peptide bond, that of the N-terminal side, and the second peptide bond, that of the C-terminal side. For Fa A, the lowest nπ*_CO_ excited state was localized on the first peptide bond, whereas for Fa B-D, which exhibits a folded backbone, the lowest nπ*_CO_ excited state was localized on the second peptide bond. The fifth state for Fa A-C corresponded mainly to an nπ* charge transfer (CT) excited state involving an electronic charge transfer from the lone pairs of the backbone (one or two peptide bonds) to the phenyl ring. In the case of Fa D, the fifth state was mainly a locally nπ*_CO_ excited state (74%), and the first state with a dominant CT character was the ninth state.

**LC-ωPBE:** The nature of the four low-lying excited states was well reproduced for all the four conformers. However, among the ten lowest low-lying excited states, no CT excited state was identified for any of the four conformers, and only states with minor CT contributions were identified. In particular, the fifth excited state corresponded to a pure locally ππ* excited state centered on the phenyl ring for Fa C and mainly to a ππ* excited state (58%, 48%, and 55%) for Fa A, B, and D (Table S3a–d). We decided then to study the influence of the ω parameter of the functional on the existence of CT states and, in particular, on the CT contribution in these low-lying excited states. In order to achieve this, we performed a series of calculations at different values of ω for A and B conformers: the standard value 0.4 bohr^−1^ and four surrounding values, i.e., 0.45, 0.35, 0.30, and 0.25 bohr^−1^. The results highlighted that a ω value of 0.30 bohr^−1^ was the best compromise allowing to obtain for the fifth excited state a CT contribution in accordance with the CC2 data, without damaging the nature of the other valence excited states, in particular the fourth state, which corresponded to a ππ* excited state (Table S3e). However, the mean absolute error (MAE) between the CC2 and TD-DFT levels on the vertical excitation energies of these valence states significantly increased for this ω value (Table S3f).

**CAM-B3LYP and ωB97X-D:** The nature of all excited states (Tables S4a–d and S5a–d) were well reproduced by the two functionals except the fifth state of Fa D, which corresponded mainly to a CT state, in contrast with the locally nπ*_CO_ excitation feature found at the CC2 level. The mean absolute (MAE) and mean signed errors (ME) on the vertical excitation energies were similar: 0.21 and −0.13 eV, respectively, for CAM-B3LYP and 0.19 and −0.10 eV, respectively, for ωB97X-D. In addition, if the vertical excitation energies of the lowest ππ* excited states were overestimated (an MAE of 0.19 eV with an ME of +0.19 eV for both functionals), all the others excited states including the higher ππ* excited states were underestimated (an MAE and ME of 0.24 and −0.24 eV, respectively, for CAM-B3LYP; 0.19 eV and −0.19 eV, respectively, for ωB97X-D).

#### 2.1.2. Geometry Optimization of the Lowest Singlet ππ*, IR Signature, and 0–0 Excitation Energy

In view of the poor results obtained on the nature and vertical excitation energies for the Fa A-D five lowest excited states with the LC-ωPBE functional, the geometry optimization of the lowest excited state (S_1_), a ππ* excited state, was only performed with the CAM-B3LYP and ωB97X-D functionals.

**Figure 3:**
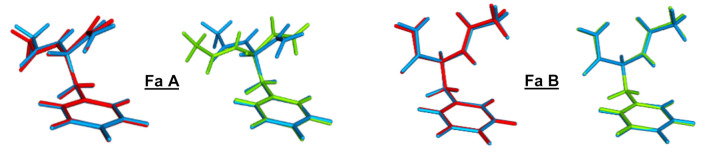
Optimized geometries of the S_1_ state for Fa A and B. Comparison of the CC2/cc-pVDZ geometry (blue structures) with that obtained with ωB97XD/cc-pVDZ (red structures) and with CAM-B3LYP/cc-pVDZ (green structures). For the sake of comparison, phenyl rings were overlapped.

Selected characteristic geometrical parameters of the lowest ππ* excited state optimized geometry of Fa A-D are reported in Table S6a–c for the CC2, CAM-B3LYP, and ωB97X-D levels, and a comparison of the whole set of geometries is depicted in Figure 3 and Figure S6. Whereas only relatively small deviations from CC2 were obtained with the ωB97X-D functional for the four Fa A–D conformers, the CAM-B3LYP functional gave a significant deviation for Fa A. Indeed, the interactions that govern the arrangement of the backbone above the phenyl ring and, more particularly, the NH…π interaction differed strongly in this conformer as if the dispersion forces were not quite correctly taken into account by the CAM-B3LYP functional. In fact, the NH_2_ group was shifted out of the phenyl ring, and the NH…π interaction was strongly reduced. With the ωB97X-D functional, all the discrepancies for the Fa A-D optimized geometries fell within a range from −8 to 6° for the dihedral angles with a mean absolute deviation (MAD) of 3° and a range from −0.04 to 0.14 Å for the intramolecular distances with a MAD of 0.05 Å. Similar discrepancies were obtained with CAM-B3LYP for the Fa B–D conformers, i.e., within a range from −8 to 6° for the dihedral angles with a MAD of 4° and a range from −0.06 to 0.23 Å for the intramolecular distances with a MAD of 0.10 Å. On the contrary, the geometry of the Fa A exhibited large discrepancies, i.e., into a range from 4 to 26° for the dihedral angles with a MAD of 17° and into a range from −0.43 to 0.75 Å for the intramolecular distances with a MAD of 0.33 Å.

On the contrary, the optimized geometry of the ground state of Fa A (S_0_, see Table S6a–c) obtained with CAM-B3LYP did not present such large deviations. The discrepancies from 0 to 5° for the dihedral angles with a MAD of 5° and from −0.05 to 0.12 Å for the intramolecular distances with a MAD of 0.07 Å, were similar to those obtained for Fa B–D ground states, i.e., from −9 to 5° for the dihedral angles with a MAD of 4° and from −0.06 to 0.22 Å for the intramolecular distances with a MAD of 0.11 Å. Furthermore, they were comparable to those obtained with ωB97X-D for the Fa A–D ground states, i.e., from −8 to 2° for the dihedral angles with a MAD of 2° and from −0.04 to 0.11 Å for the intramolecular distances with a MAD of 0.05 Å. These results led to a root mean square deviation (RMSD) of the ωB97X-D intramolecular distances relative to CC2 for the Fa A–D ππ* excited state (S_1_) conformers almost equal to that obtained for the Fa A–D ground state (S_0_) conformers, i.e., 0.06 Å and 0.07 Å, respectively. These very close values were small but larger by one order of magnitude than those obtained for the covalent bonds. Moreover, no difference was observed between RMSD in the ground (S_0_) and in the first ππ* excited state (S_1_). This was in contradiction with what was reported for the covalent bond lengths by us in this work as well as by others in a benchmark set of 20 medium-sized aromatic organic compounds [37] for which the RMSD(S_1_) for these two functionals, despite being small, 0.009 Å, was larger than their RMSD(S_0_), i.e., 0.006 Å. These results demonstrate that non-covalent bonds governed by relatively weak interactions, such as dispersion forces, were more sensitive to the method than the covalent bonds but, on the other hand, do not depend significantly on the nature of the state, ground or excited.

In order to go further in the comparison of geometries, the harmonic vibrational frequencies, in particular, the three NH stretch frequencies (NH_Phe_, NH_2sym_, and NH_2anti._) of the *amide A* region (Table S7) and their shifts between the S_1_ and the S_0_ states (Table 2) were calculated. Once again, except for Fa A with the CAM-B3LYP functional, the CC2 values were relatively well reproduced by the two functionals, especially if we first consider the order of magnitude as well as the sign of the shift. Indeed, except for CAM-B3LYP Fa A, when a large experimental shift was obtained, the two functionals gave large theoretical shifts close to that obtained by the CC2 method, and a similar behavior was obtained for small shifts. Regarding the comparison with the experiment, DFT/TD-DFT and CC2 predict the shift sign properly but tend to overestimate the frequency shifts, in line with a general overestimate of the harmonic frequencies in the *amide A* region. We then carried out for the series of capped peptides (see the following section) the protocol developed for the CC2 calculations in order to obtain the mode-dependent corrected frequencies from the optimal harmonic frequency mode-dependent (NH_Phe_, NH_2sym_, and NH_2anti_) linear scaling functions [18].

Finally, if the conformers stability order compared to both CC2 and experiment (Table 3 and Table 4) was well reproduced by the two functionals, we observed a systematic similar strong overestimation of the adiabatic ZPVE-corrected excitations energies. A MAE of 0.40 eV (0.41) with a ME of +0.40 (0.41) eV for ωB97X-D (CAM-B3LYP) compared to CC2 and a MAE of 0.50 eV (0.51) with a ME of +0.50 (+0.51) eV for ωB97X-D (CAM-B3LYP) compared to the experiment were obtained whereas the MAE (ME) for CC2 [18] compared to experiment was of 0.11 (+0.11) eV only. Such a similar behavior was previously reported for a set of 66 organic medium- and large-sized molecules containing aromatic heterocyclic compounds and aromatic (aliphatic) hydrocarbons as well as substituted aromatic hydrocarbons with (MAE, ME) of (0.32, +0.30) eV for ωB97X-D/def2-TZVP ((0.33, +0.30) for CAM-B3LYP)/def2-TZVP) compared to experiment, whereas the CC2/def2-TZVP (MAE, ME) was (0.11, +0.09) eV [33].

### 2.2. 0–0 Excitation Energies and IR Signature of the ππ* for the Series of Capped Peptides

The results obtained on the reference system, the Fa A-D conformers, in particular, those highlighting that the dispersion forces are only partially taken into account with CAM-B3LYP, led us to select only the ωB97X-D functional in order to perform the calculations on the series of capped peptides.

**Figure 4:**
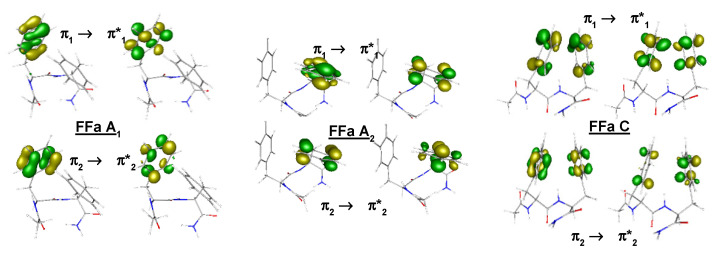
Couples of NTOs at the ωB97XD/cc-pVDZ level for FFa A_1_, A_2_, and C. Only couples whose contribution to the wave function is greater than 10% are drawn (see Table S9 for the values of contribution).

First of all, as for the reference system, the nature and the optimized geometry of the lowest ππ* excited state were well reproduced compared to those obtained at the CC2 level (Tables S8a–c and S9). In particular, FFa A led to two low-lying ππ* excited states FFa A_1_ and FFa A_2_, for which the excitations were localized on either of the phenyl rings whereas FFa C led to one low-lying ππ* excited state for which the excitation was delocalized on the two phenyl rings (Figure 4 and Figure S9 and Table S9). Moreover, these qualitative differences were in perfect adequacy with the experimental data [18]. Finally, the MAD obtained for the characteristic geometrical parameters of S_0_/S_1_ states were very similar to that obtained for the reference system, 3–4° for the dihedral angles and 0.04–0.05 Å for the intermolecular distances to compare to 3–2° and 0.05–0.05 Å.

Regarding the energetics (Table 4 and Table 5) and compared to CC2, a systematic strong overestimation of the adiabatic ZPVE-corrected excitation energies (MAE of 0.41 eV with an ME of +0.41 eV) similar to that obtained for the reference system was observed for all the conformers except that of FFa C, which was underestimated (−0.26 eV). The total MAE (reference system plus the series of capped peptides) was equal to 0.39 eV with a total ME of +0.35 eV. A similar strong overestimation was observed when we compared to the experiment: a total MAE of 0.50 eV with a total ME of +0.43 eV. Again, the FFa C adopted a behavior opposite to that of all the other conformers, and its adiabatic ZPVE-corrected excitation energy was strongly underestimated at 0.41 eV. In this context, it is important to remind that this exception concerns the FFa C conformer for which the 0−0 transition was not directly measured but extrapolated by comparison with the vibronic progression of an analogous system containing only one phenylalanine [18]. Finally, the errors at the ωB97X-D level on the ZPVE-corrected adiabatic excitations energies compared to both CC2 and experiment seems systematic along with the series without any significant size effects.

The harmonic and mode-dependent corrected frequency shifts of the NH stretch vibrations between the ground and the ππ* excited states were calculated for all the conformers and reported in Table 6 together with those at the CC2 level and the experimental ones when available. The mode-dependent corrected shifts have been calculated from the mode-dependent corrected frequencies, which have been determined from the optimal harmonic frequency mode-dependent (NH, NH_2sym_, and NH_2anti_) linear scaling functions (ν_exp._ = aν_theo._+ b). The linear scaling functions were determined from both the 42 experimental *amide A* region frequencies available for S_0_ and the 22 experimental *amide A* region frequencies available for S_1_ (Tables S7a and S10b,d,f) and allowed us to take into account in these theoretical S_0_ and S_1_ frequencies both the method and basis set errors and anharmonicity effects (Figure S11). As followed for the CC2 level, this strategy of systematic correction of calculated harmonic frequencies provides reliable predictions of the theoretical S_1_-S_0_ frequency shifts in the *amide A* region for such systems, with an RMSD of 6 cm^−1^ similar to that obtained at the CC2 level, i.e., 5 cm^−1^ [18].

### 2.3. Composite Protocols CC2-DFT/TDDFT and 0–0 Excitation Energies Calculations

The adiabatic ZPVE-corrected excitation energies were calculated according to two protocols for the full series of capped peptides (Table 4 and Table 7). These two protocols combined DFT/TD-DFT and CC2 calculations. In the first protocol denoted hereafter P1, both the geometry optimizations and harmonic frequencies calculations were performed at the DFT/TD-DFT level. In the second protocol, P2, the geometry optimizations were performed at the CC2 level, whereas the harmonic frequencies calculations were performed at the DFT/TD-DFT level (see Methods section for details). A systematic overestimation was observed except for FFa C, like what was obtained at the CC2 level. The two protocols led to a decrease in the error compared to both CC2 and the experiment. However, this decrease was moderate for protocol P1, whereas protocol P2 exhibited a strong decrease, eventually providing an error compared to the experiment similar to that obtained at the CC2 level. Indeed, P1 gives an MAE of 0.15 eV with an ME of +0.14 eV compared to CC2 and an MAE of 0.33 eV with an ME of +0.22 eV compared to the experiment, whereas P2 gives an MAE of 0.03 eV with an ME of +0.02 eV compared to CC2 and an MAE of 0.14 eV with an ME of +0.11 eV compared to the experiment. In addition, as for the CC2 method, no size effects were observed for the two protocols. Indeed, in the P1 protocol, the MAE for the capped peptides containing one residue was equal to 0.20 eV (ME of +0.20 eV) compared to CC2 and 0.31 eV (ME of +0.31 eV) compared to the experiment, whereas the MAE for the capped peptides containing two residues was equal to 0.23 eV (ME of +0.10 eV) compared to CC2 and 0.34 eV (ME of +0.17 eV) compared to experiment. The protocol P2, compared to CC2, provides an MAE of 0.02 eV (ME of +0.02 eV) for the capped peptides containing one residue and an MAE of 0.03 eV (ME of +0.02 eV) for the capped peptides containing two residues.

The difference between MAE and ME for the systems containing two residues is essentially due to the underestimation obtained for FFa C. In addition, a similar behavior was observed for the comparison with the experiment: the MAE was equal to 0.14 eV (ME of +0.14 eV) for the capped peptides containing one residue and equal to 0.14 eV (ME of +0.09) for the capped peptides containing two residues. The protocol P2 allows then to reproduce very well the CC2 adiabatic ZPVE-corrected excitation energies and then to obtain a quantitative agreement on these data compared to the experiment as for the CC2 method. This very good agreement can be explained by the low sensitivity of the $\Delta \Delta E_{00}$ term, the difference of ZPVE corrections between the ground (S_0_) and excited (S_1_) states relative to the method, CC2 or DFT/TD-DFT.

Such behavior was already reported by Durbeej et al. for the set of 66 organic medium-sized and large molecules containing aromatic heterocyclic compounds and aromatic (aliphatic) hydrocarbons as well as substituted aromatic hydrocarbons [34]. Finally, these two protocols give results that, compared to the experiment, behave identically to the CC2 ones, i.e., systematic overestimation and no size effect. They can, therefore, be a reliable alternative to the CC2 method for large and very large-sized systems with two levels of accuracy, a high level with P2 and a moderate one with P1.

## 3. Methods

### 3.1. Benchmark Set Composition

The benchmark set (Figure 1) consists of the four conformers (A–D) of a reference system, *N*-acetyl-phenylalaninylamide (Ac-Phe-NH_2_, Phe: phenylalanine), and two conformers of three capped dipeptides, Ac-Gly-Phe-NH_2_ (A and B’, Gly: glycine), Ac-Phe-Phe-NH_2_ (A and C) and Ac-Gln-Phe-NH_2_ (A and C, Gln: glutamine). These systems adopt in their ground state prototypical secondary structural features of proteins [15] and present specific low-lying excited states, such as localized or delocalized states on the different significant groups of these systems (aromatic rings and peptide bonds) as well as charge transfer states from the backbone to the phenyl ring. Detailed information regarding these systems is summarized below. The L label refers to the preferential backbone orientation adopted by the natural amino acids of L configuration, as opposed to those of D configuration. The labels a, g+, or g- refer to the anti, gauche+, or gauche- orientation of the phenyl side chain relative to the backbone, the χ_i_^1^ dihedral angle N−C_α_-C_β_-C_γ_ being close to +180° for a, +60° for g+, and −60° for g- (Figure S1b). Finally, the index n of the H-bond label C_n_ indicates the number of atoms in the ring formed by the H-bond.

**The reference system, Fa**:[16,18,36,38] In the ground state, four conformers (A–D) with two different types of folding backbone were observed and assigned to conformations lying in an energy range of 0−6.5 kJ/mol: one β-strand extended conformation (A (β_L_(a)) stemming from a C_5_ H-bond and three γ-turn folded conformations stabilized by a C_7_ H-bond and differing by the orientation of the phenyl side chain (B (γ_L_(g+)), C(γ_L_(g-)) and D(γ_L_(a))). Three structures present an NH···π bond, the β-strand conformation A, and two γ-turns, B and C. The corresponding 0–0 excitation energy to the lowest ππ* excited state was both theoretically and experimentally determined for each conformer, whereas only excited state IR (ESIR) spectra of A and C were measured.

**GFa**:[15,16,18,38] Among the five conformers observed and assigned to conformations lying in an energy range of 0−6 kJ/mol, two conformers were chosen, A and B’. They correspond to two different types of folding backbone: a 7-7 (double γ-turn) extended conformation with an L chirality of the turns (A (7_L_-7_L_(g-)) and a β-turn folded conformation of types II’ (B’(π-10II’(g+)). These two families of backbone folding correspond to secondary structural features of proteins: the 2_7_ ribbon and β-turn secondary structures, respectively stabilized by successive C_7_ H-bonds (γ-turns) and C_10_ H-bonds (β-turn). In addition, one structure, the β-turn, presents an NH…π bond. The corresponding 0-0 excitation energy to the lowest ππ* excited state was both theoretically and experimentally determined for each conformer as well as their ESIR spectra.

**FFa**:[16,18,39] Among the three conformers observed and assigned to conformations lying in an energy range of 0−15 kJ/mol, two conformers, A and C, were chosen. They correspond to both different types of backbone folding and different relative orientations of the two phenyl rings: one β-turn type I conformation (an α_L_-γ_L_ structure, A (π-π-10I(g+,g+))) where the phenyl rings interact according to a T-shape arrangement and one β_L_-γ_L_ conformation (C (5-π-7_L_(a,g+))) where the phenyl rings interact according to a face-to-face arrangement. FFa A is characterized by a C_10_ H-bond and C by a usual combination of C_5_ and C_7_^eq^ H-bonds. In addition, these conformations present at least one NH-π bond. For the A conformer, the two intense transitions observed in the near UV spectrum were assigned, in accordance with the CC2 calculations and ESIR spectra, to the origin transition of each excited chromophore, A_1_ and A_2_. On the contrary, the experiment suggested that the C conformer exhibits only one intense transition in the near UV spectrum, which can be assigned to the lowest ππ* excited state. CC2 calculations confirmed this hypothesis showing that this excited state was delocalized on the two chromophores. Only ESIR spectra of A_1_ and A_2_ were measured.

**QFa**:[18,40] Among the three conformers observed and assigned to conformations lying in an energy range of 0−7.5 kJ/mol, two conformers were chosen, A and C. They corresponded to the same type of folding backbone, i.e., a type I β-turn backbone, stabilized by a C_10_ H-bond. This bond was combined to a side chain/main chain C_7_ H-bond bridging the NH site of the first peptide bond to the oxygen atom of the Gln residue side chain CO-NH_2_ group labeled 7^ε^ and presenting a similar NH…π bond; A (7^ε^-π-10I(g+)) and C (7^ε^-π-10I(g+)). The two structures differed by the orientation of the CO-NH_2_ group, i.e., the conformation of the Gln residue side chain. The corresponding 0–0 excitation energy to the lowest ππ* excited state was determined both theoretically and experimentally for both conformers.

### 3.2. DFT/TD-DFT Calculations

DFT/TD-DFT calculations were carried out with the Gaussian Package [41]. Ground-state DFT and excited-state TD-DFT calculations were performed by using three long-range corrected hybrid functionals, LC-ωPBE [28], CAM-B3LYP [29], and ωB97X-D [30]. The difference between these long-range hybrid GGA functionals resides mainly in the percentage of short and long-range exact exchange and the explicit taken into account of a dispersion correction. The CAM-B3LYP functional comprises 19% exact exchange plus 81% Becke 1988 (B88) exchange interaction at short-range, and 65% exact exchange plus 35% B88 at long-range, the intermediate region being smoothly described through the standard error function with parameter 0.33. In the LC-ωPBE functional, the short-range and long-range exact exchange coefficient is equal to 1 with an ω equal to 0.40 bohr^−1^. The ωB97X-D functional includes 22.2% short-range exact exchange and 100% long-range exact exchange controlled by an *ω* of 0.20 bohr^−1^. In addition, this functional includes explicitly a dispersion correction via empirical potentials of the form −C_6_/R^6^. In order to compare to our previous CC2 calculations,[18] we used the same combination of basis sets: (i) the Dunning’s correlation-consistent polarized valence double-ξ (cc-pVDZ) basis set [42] for single-point energy calculations at the ground state of the reference system, the geometry optimizations of both ground state and the first ππ* excited states of the benchmark set as well as the harmonic frequencies calculations; (ii) this basis set was augmented with diffuse functions taken from the aug-cc-pVDZ [43] on each oxygen and nitrogen atom and only for one in two carbon atoms of the phenyl ring for the single-point energy calculations. Our previous work demonstrated on a series of capped peptides of increasing size and containing different residues that the CC2 method in combination with the aug(N,O,π)-cc-pVDZ//cc-pVDZ basis sets, proves to be very reliable for calculations of the 0-0 excitation energies of such systems compared to experiment, leading to a mean absolute error (MAE) of 0.10 eV and a mean signed error (ME) of +0.08 eV. In addition, this level of theory was further justified by the faster convergence of DFT/TD-DFT calculations, with respect to the size of the one-electron basis set, compared to conventional correlated methods such as CC2. All DFT and TD-DFT calculations were performed using the *ultrafine* grid. In the ground-state DFT calculations, the convergence criterion on the RMS density was 10^−8^. In the TD-FDT calculations, fifteen states were solved, and the convergence threshold was 10^−3^ au on the wave function and 10^−6^ au on the energy for single-point energy calculations and 10^−6^ au on the wave function and 10^−8^ au on the energy for geometry optimizations. The geometry of both ground and excited states was optimized until the residual mean force was smaller than 4.5 10^−4^ au, and the harmonic frequencies were analytically (DFT) or numerically (TD-DFT) determined. Harmonic frequencies calculations allow us to both verify that the optimized geometries correspond to true minima and calculate for each conformer and state the zero-point vibrational energy (ZPVE). In addition, taking advantage of a large amount of experimental data available (42 *amide A* region frequencies available for S_0_ and 22 for S_1_), we determined optimal harmonic frequency mode-dependent (NH, NH_2sym_, and NH_2anti_) linear scaling functions (ν_exp._ = aν_theo._+ b) in order to correct the calculated S_0_ and S_1_ harmonic frequencies for method and basis set errors as well as anharmonicity effects.

### 3.3. Composite CC2/DFT-TDDFT Protocols for 0-0 Excitation Energies

We evaluated two composite protocols to calculate the adiabatic ZPVE-corrected excitation energies (ΔE_00_) according to the approach developed in ref. [32]. In the first one, denoted hereafter P1, the CC2 [21,22,23,24,25] method was only used to calculate the vertical excitation energy calculation (ΔE_v_), and both the geometry optimizations and harmonic frequencies calculations were performed with the DFT(S_0_) and TD-DFT(S_1_) methods. In the second protocol (P2), the CC2 method was used for both the vertical excitation energy calculation and the geometry optimizations, but the harmonic frequencies calculations were performed with the DFT(S_0_) and TD-DFT(S_1_) methods:

$$\Delta E_{00}=\Delta E_{v}+\Delta \Delta E_{\mathrm{ad}}+\Delta \Delta E_{00}$$

$$\begin{array}{c} \Delta \Delta E_{\mathrm{ad}}=\Delta E_{\mathrm{ad}}-\Delta E_{v}\\ \Delta \Delta E_{00}=\Delta E_{00}-\Delta E_{\mathrm{ad}} \end{array}$$

$$\begin{array}{c} P1:\;\Delta E_{00}=\Delta E_{v}(\mathrm{CC}2)+\Delta \Delta E_{\mathrm{ad}}(\mathrm{DFT}/\mathrm{TD}-\mathrm{DFT})+\Delta \Delta E_{00}\left(\mathrm{DFT}/\mathrm{TD}-\mathrm{DFT}\right)\\ P2:\;\Delta E_{00}=\Delta E_{v}(\mathrm{CC}2)+\Delta \Delta E_{\mathrm{ad}}(\mathrm{CC}2)+\Delta \Delta E_{00}\left(\mathrm{DFT}/\mathrm{TD}-\mathrm{DFT}\right) \end{array}$$

where ΔE_v_ is the vertical excitation energy at the equilibrium geometry of the ground-state, ΔE_ad_ is the energy difference between the ground and excited states at their respective optimized geometry, and ΔE_00_ is this energy difference, including the zero-point vibrational energy (ZPVE) corrections. A combination of two basis set was used for all the calculations. First, the geometry optimizations and frequency calculations were performed within the cc-pVDZ basis set, whatever the method used, and second, the single-point energy calculations for the determination of the vertical excitation energies were performed within the aug(N,O,π)-cc-pVDZ.

The CC2 computational details summarized hereafter were those of our previous benchmark [18]. CC2 calculations were performed with the TURBOMOLE package [44] using the resolution-of-identity (RI) approximation and associated auxiliary basis sets [45]. Frozen cores for the 1s electrons were employed, and all calculations were carried out in the *C_1_* point-group symmetry. Ten singlet states were considered, and D_1_, D_2_ diagnostics and % $<\bar{E_{1}}|E_{1}>$ biorthogonal norm were calculated in order to evaluate the capability of the CC2 method to properly describe the ground and excited states of such systems [25,46,47]. The convergence criterion used in single-point energy calculation was 10^−8^ au on the density for the HF calculation, 10^−9^ au for the RI-CC2 ground state energy for the iterative coupled-cluster methods, and 10^−6^ au for the convergence threshold for the norm of residual vectors in eigenvalue problems for the RI-CC2 excited states calculations. In the geometry optimization of both ground and lowest ππ* excited states, the convergence criterion used corresponded to a norm of the Cartesian gradient lower than 10^−4^ au. Finally, the harmonic frequencies were calculated by numerical differentiation of the analytic gradients using central differences and a step length of 0.02 au.

### 3.4. Characterization of the Excited States

Orbital-relaxed first-order properties were determined, in particular the density, at DFT or CC2 level for the ground states and at TD-DFT or CC2 level for the excited states. DFT/TD-DFT and CC2 differences between the density of excited states and that of the ground state were then calculated. In addition, a post-processing tool interfaced to TURBOMOLE and GAUSSIAN, Nancy_EX-2.0 [48], was used in order to analyze the density and character of the excited states and obtain, at both TD-DFT and CC2 level, the so-called natural transition orbitals (NTOs) [49,50] of each state. Instead of describing one excitation with multiple canonical spin orbitals couples, all the physical information on the nature of the electronic transition was gathered in one (sometimes two) couple(s) of NTOs.

## 4. Conclusions

Benchmarking calculations were performed to determine the ability of the TD-DFT method to model qualitatively the PES of bio-relevant systems such as capped peptides. In the first step, the most accurate functional, ωB97X-D, among three long-range corrected hybrid functionals have been selected on criteria based on energetics as well as the first and second derivatives of the energy of the low-lying excited states of the four conformers of the smallest capped peptide, Fa A–D. This highlights that the intramolecular dispersion interactions can play an important role in the geometry of prototypical secondary structures of proteins and must be taken into account explicitly. In the second step, this functional was validated on a series of capped peptides of increasing size and containing residues of different nature. We obtained for the geometry optimization of both ground and ππ* excited states as well as for the harmonic frequencies calculations a quantitative agreement compared to CC2 and experiment. However, the adiabatic ZPVE-corrected excitation energies were systematically strongly overestimated, and we then developed protocols combining CC2 and DFT/TD-DFT methods, and one of these protocols allowed us to obtain excitation energies of CC2 quality at the lowest computational cost (Table S12).

In conclusion, the TD-DFT method with a system-appropriate functional, the ωB97X-D for capped peptides, is well suited for a qualitative exploration of the PES of the low-lying excited states, a key step in non-adiabatic dynamics simulations performed to determine deactivation mechanisms in bio-relevant systems. In addition, computation of adiabatic ZPVE-corrected excitation energies of CC2 quality can be obtained by employing CC2 for both the single-point energy calculations (vertical excitation energies) and the geometry optimizations and DFT/TD-DFT for the calculation of the ZPVE corrections. We even obtained a moderate agreement using the CC2 method only for the single-point energy calculations and the DFT/TD-DFT for both the geometry optimizations and frequencies calculations. These protocols, therefore, constitute an alternative to the CC2 method for very large systems, i.e., when this computationally demanding method (see Table S12) is no longer applicable. Finally, the TD-DFT method exhibits a favorable cost-performance ratio, and its involvement in the calculations can be adapted according to the level of expected accuracy.

## Figures and Tables

**Table 1:** Nature and CC2/cc-pVDZ vertical excitation energies of the five lowest excited states of Fa B at the B97-D2/TZVPP optimized geometry of the ground state.

Fa B	E_vert_^[a]^ (eV)	Nature of the State	NTOs (%, Occupied→Virtual) ^[b]^
S_1_	5.259	ππ*	**1**–53% 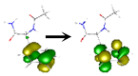 **2**–47% 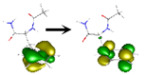
S_2_	5.781	nπ*_CO(2)_	**1**–98% 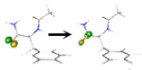
S_3_	5.905	nπ*_CO(1)_	**1**–99% 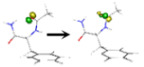
S_4_	6.534	ππ*	**1**–73% 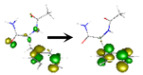 **2**–25% 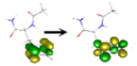
S_5_	6.787	n_(1,2)_ [π*π*_CO(1)_] + ππ*	**1**–80% 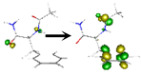 **2**–12% 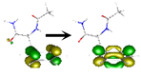

^[a]^ The theoretical values are given with the number of significant digits obtained in the experiment. ^[b]^ In the event of several couples of natural transition orbitals (NTOs), only those whose contribution to the wave function is greater than 10% are reported.

**Table 2:** Theoretical shifts (harmonic x and mode-dependent corrected y, i.e., x or x/y) of the *amide A* region frequencies of the lowest ππ* excited state optimized geometry (S_1_) relative to the ground state optimized geometry (S_0_) for the Fa A–D conformers obtained at the CC2/, CAM-B3LYP and ωB97X-D/ /cc-pVDZ levels, together with the corresponding available experimental data (cm^−1^).

	Δν_S1/S0_ (cm^−1^)	CC2 [17]	CAM-B3LYP	ωB97X-D	Experiment
Fa A	NH_Phe_	+11/+9	−6	+10	−1
	NH_2 sym._	−15/−10	−29	−9	−9
	NH_2 anti._	−18/−9	−22	−8	−6
Fa B	NH_Phe_	−44/−37	−44	−25	
	NH_2 sym._	−5/−4	−5	−6	
	NH_2 anti._	−1/−1	−2	−3	
Fa C	NH_Phe_	−44/−37	−38	−44	−24
	NH_2 sym._	−3/−1	−8	−2	−1
	NH_2 anti._	−1/−1	−1	+2	−1
Fa D	NH_Phe._	0/0	−1	−1	
	NH_2 sym._	−2/−2	0	−7	
	NH_2 anti_	0	9	−1	

**Table 3:** Adiabatic ZPVE-corrected excitation energies of the lowest ππ* excited state (S_1_) of the Fa A-D conformers obtained at the CC2/aug-(N,O,π)-cc-pVDZ//CC2/cc-pVDZ, the CAM-B3LYP/aug-(N,O,π)-cc-pVDZ//CAM-B3LYP/cc-pVDZ and ωB97X-D/aug-(N,O,π)-cc-pVDZ//ωB97X-D/cc-pVDZ levels, together with the experimental 0–0 transition energies.

ΔE_adia_ (eV)	Fa A	Fa B	Fa C	Fa D
CC2/aug(N,O,π)-cc-pVDZ//CC2/cc-pVDZ [17]	4.754	4.770	4.767	4.791
CAM-B3LYP/aug(N,O,π)-cc-pVDZ//CAM-B3LYP/cc-pVDZ	5.168	5.167	5.142	5.192
ωB97XD/aug(N,O,π)-cc-pVTZ//ωB97XD/cc-pVDZ	5.140	5.161	5.160	5.174
Experiment ^[a]^	4.650	4.663	4.653	4.666

^[a]^ The experimental values take into account the number of significant digits obtained in the experiment, and the theoretical values are given with the same number of digits.

**Table 4:** MAE and ME (eV) on the adiabatic ZPVE-corrected excitation energies of the lowest ππ* excited state (S_1_) between DFT/TD-DFT, P1 or P2 and CC2 or Experiment. For DFT/TD-DFT, only the results obtained with (ωB97X-D) are reported. For the definition of P1 and P2, see the following section and Methods section. Bs designed all the systems contained in the benchmark set (see Methods section).

Calculation Systems		MAE	ME	MAE	ME
Level		CC2	CC2	Exp.	Exp.
DFT/TD-DFT	Fa A-D	0.40	+0.40	0.50	+0.50
DFT/TD-DFT	Bs	0.39	+0.35	0.50	+0.43
P1	Bs	0.15	+0.14	0.33	+0.22
P2	Bs	0.03	+0.02	0.14	+0.11

**Table 5:** Adiabatic ZPVE-corrected excitation energies of the lowest ππ* excited state (S_1_) of the GFa A and B’, FFa A_1_, A_2_ and C and QFa A and C conformers obtained at the CC2/aug-(N,O,π)-cc-pVDZ//CC2/cc-pVDZ and ωB97X-D/aug-(N,O,π)-cc-pVDZ//ωB97X-D/cc-pVDZ levels, together with the experimental 0–0 transition energies.

ΔE_adia_ (eV)	GFa A	GFa B’	FFa A_1_	FFa A_2_	FFa C	QFa A	QFa C
CC2							
aug(N,O,π)-cc-pVDZ//							
cc-pVDZ [17]	4.754	4.771	4.714	4.729	4.479	4.803	4.793
ωB97XD							
aug(N,O,π)-cc-pVTZ//							
cc-pVDZ	5.178	5.178	5.154	5.149	4.219	5.207	5.162
Experiment ^[a]^	4.648	4.644	4.648	4.658	4.630	4.662	4.658

^[a]^ Same details as Table 3.

**Table 6:** Theoretical shifts (harmonic x and mode-dependent corrected y, i.e., x or x/y) of the *amide A* region harmonic frequencies of the lowest ππ* excited state optimized geometry (S_1_) relative to the ground state optimized geometry (S_0_) for the GFa A and B’, FFa A_1_, A_2_, and C and QFa A, C conformers obtained at the CC2/ and ωB97X-D(x)/cc-pVDZ levels, together with the corresponding available experimental data (cm^−1^).

	Δν_S1/S0_ (cm^−1^)	CC2 [17]	ωB97X-D	Experiment
GFa A	NH_Gly_	−7/−6	−2/−2	−2
	NH_Phe_	−18/−15	−10 */−9	−18
	NH_2 sym._	−5/−3	+3 */+2	+3
	NH_2 anti._	0/0	+1/+1	−9
GFa B’	NH_Gly_	0/0	−1/−1	+1
	NH_Phe_	−21/−18	−4/−4	−18
	NH_2 sym._	+1/+1	+1/+1	+2
	NH_2 anti._	0/0	+1/+1	+1
FFa A_1_	NH_Phe1_	−41/−35	−40/−36	−33
	NH_Phe2_	−9/−8	−7/−6	0
	NH_2 sym._	−4/−3	+3/+2	−1
	NH_2 anti._	−2/−1	0/0	0
FFa A_2_	NH_Phe1_	−5/−4	−6 */−5	−1
	NH_Phe2_	−34/−29	−21 */−19	−24
	NH_2 sym._	−2/−1	+3/+2	−1
	NH_2 anti._	−1/−1	0/0	0
FFa C	NH_Phe1_	−12/−10	−9/−8	
	NH_Phe2_	−74/−63	−60 */−55	
	NH_2 sym._	−30/−21	−21 */−15	
	NH_2 anti._	−11/−6	−7/−6	
QFa A	NH_Gln_	−2/−2	−6/−5	
	NH_Phe_	−12/−10	−22/−20	
	NH_2 sym./C-term_	+5/+3	−2/−1	
	NH_2 anti./C-term_	+8/+4	0/0	
	NH_2 sym./cChain_	−2/−1	0/0	
	NH_2 anti./Chain_	−2/−1	0/0	
QFa C	NH_Gln_	−9/−8	−9/−8	
	NH_Phe_	−16/−14	−32/−29	
	NH_2 sym./C-term_	−2/−1	−2/−1	
	NH_2 anti./C-term_	0/0	0/0	
	NH_2 sym./cChain_	−1/−1	−2/−1	
	NH_2 anti./Chain_	+1/+1	+3/+2	

* Coupled modes

**Table 7:** Adiabatic ZPVE-corrected excitation energies of the lowest ππ* excited state (S_1_) of the Fa A-D, GFa A and B’, FFa A_1_, A_2_, and C and QFa A and C conformers obtained with the protocols P1 and P2, together with the CC2/aug-(N,O,π)-cc-pVDZ//CC2/cc-pVDZ ones and the experimental 0–0 transition energies.

ΔE_adia_ (eV)	CC2 [17]	DFT/TD-DFT ωB97XD	P1	P2	Experiment ^[a]^
Fa A	4.754	5.140	4.953	4.778	4.650
Fa B	4.770	5.161	4.972	4.798	4.663
Fa C	4.767	5.160	4.971	4.793	4.663
Fa D	4.791	5.174	4.983	4.809	4.666
GFa A	4.754	5.178	4.867	4.814	4.648
GFa B’	4.771	5.178	4.979	4.802	4.644
FFa A_1_	4.714	5.154	4.962	4.771	4.648
FFa A_2_	4.729	5.149	4.954	4.773	4.658
FFa C	4.479	4.219	4.023	4.451	4.630
QFa A	4.803	5.207	5.014	4.825	4.662
QFa C	4.793	5.162	4.965	4.779	4.658

^[a]^ Same details as Table 3.

## Data Availability

The data presented in this study and not reported in the Supplementary Materials are available on request from the corresponding author.

## Supplementary Materials

The following are available online at https://www.mdpi.com/article/10.3390/ijms23020621/s1.
