# Supplementary material for: Excited States Computation of Models of Phenylalanine Protein Chains: TD-DFT and Composite CC2/TD-DFT Protocols
Source: Int J Mol Sci. 2022 Jan 6;23(2):621. doi: 10.3390/ijms23020621 (PMC8776158; doi:10.3390/ijms23020621)
Supplement: Supplementary file 1 [file ijms-23-00621-s001.zip › ijms-1489857-supplementary.pdf]

Supporting Information

Excited states computation of models of phenylalanine protein chains: TDDFT and composite CC2/TDDFT protocols

Marine Lebel , Thibaut Very †, Eric Gloaguen, Benjamin Tardivel, Michel Mons and Valérie Brenner*

LIDYL, CEA, CNRS, Université Paris-Saclay, 91191, Gif-sur-Yvette, France
* Correspondence: valerie.brenner@cea.fr
† Present address: IDRIS - CNRS, Orsay cedex, France, F-91403

S1: B97-D2/TZVPP optimized geometries of Fa A-D.

S2: Nature and CC2/cc-pVDZ vertical excitation energies of the five lowest excited states of Fa A, C and D at the B97-D2/TZVPP optimized geometry of the ground state.

S3: Nature and LC-ωPBE/cc-pVDZ vertical excitation energies of the five lowest excited states of Fa A-D at the B97-D2/TZVPP optimized geometry of the ground state. Variation of the nature and vertical excitation energies of the five lowest excited states of Fa B according the value of ω.

S4: Nature and CAM-B3LYP/cc-pVDZ vertical excitation energies of the five lowest excited states of Fa A-D at the B97-D2/TZVPP optimized geometry of the ground state.

S5: Nature and ωB97X-D/cc-pVDZ vertical excitation energies of the five lowest excited states of Fa A-D at the B97-D2/TZVPP optimized geometry of the ground state.

S6: Characteristic geometrical parameters of the lowest excited state (S_1) of the four Fa A-D conformers and comparison of their CC2/cc-pVDZ optimized geometries[3] with those obtained at the TD-DFT level.

S7: *Amide A* region frequencies of both the ground (S_0) and ππ* excited (S_1) states of the four Fa A-D conformers.

S8: Characteristic geometrical parameters of CC2/[3] and ωB97XD/cc-pVXZ optimized geometries of both the ground (S_0) and lowest ππ* excited (S_1) states of the GFa A, FFa A_1, A_2,C and QFa A, C conformers.

S9: NTOs of the lowest ππ* excited states (S_1) of GFa A-B, FFa A_1,A_2,C and QFa A,C at their CC2 and ωB97XD/cc-pVDZ optimized geometries.

S10: A*mide A* region frequencies of both the ground (S_0) and ππ* excited (S_1) states of the GFa A-B, FFa A_1,A_2,C and QFa A,C conformers.

S11: Experimental vs ωB97XD/cc-pVDZ calculated harmonic *amide A* region frequencies of both the S_0 and S_1 states of the series of capped peptides and the corresponding mode-dependent linear ($\nu_{exp.} = a\nu_{theo.} + b$) scaling functions.

S12: Timing of TD-DFT and CC2 calculations

References

S1: B97-D2/TZVPP optimized geometries of Fa A-D.

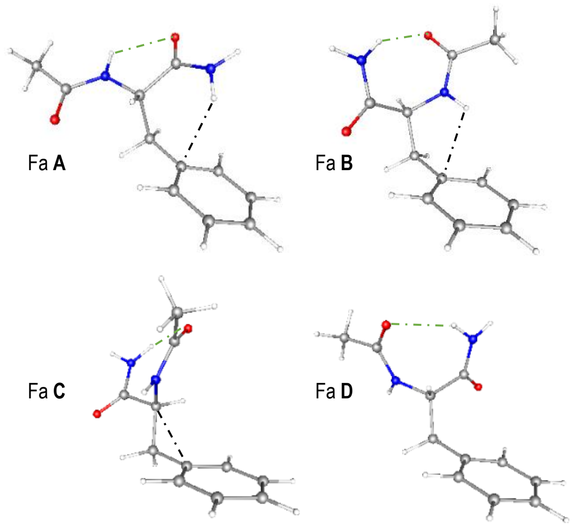

Figure S1a. B97-D2/TZVPP optimized geometries of the ground state of Fa A-D exhibiting C_5 or C_7 H-bond (green dashes) and sometimes NH…π interaction (black dashes).

Table S1. Characteristic geometrical parameters of the ground state (S_0) of Fa A-D optimized at the DFT-D level.[1-2]

		Dihedral angles (°)[a]			Intermolecular distances (Å)	
		Φ	Ψ	χ^1	$d_{NH...O}$	$d_{NH...\pi}$[b]
S_0	A	-160	159	192	2.28	2.56 (3.37, 2.84)
	B	-83	55	44	2.02	2.44 (3.23, 2.54)
	C	-85	72	-55	2.03	2.77 (3.76, 2.85)
	D	-83	84	193	2.24	

[a] For the definition of the dihedral angles, see Figure S1b.
[b] The NH…π bond is characterized by three distances: the distance of the NH_2 (A conformer) or NH_{Phe} (B conformer and C conformer) hydrogen atom with the C_γ carbon atom of the phenylalanine residue and in parentheses the two distances with the two C_δ carbon atoms ($C_\delta^{C\text{-term}}$, $C_\delta^{N\text{-term}}$) of the phenylalanine residue.

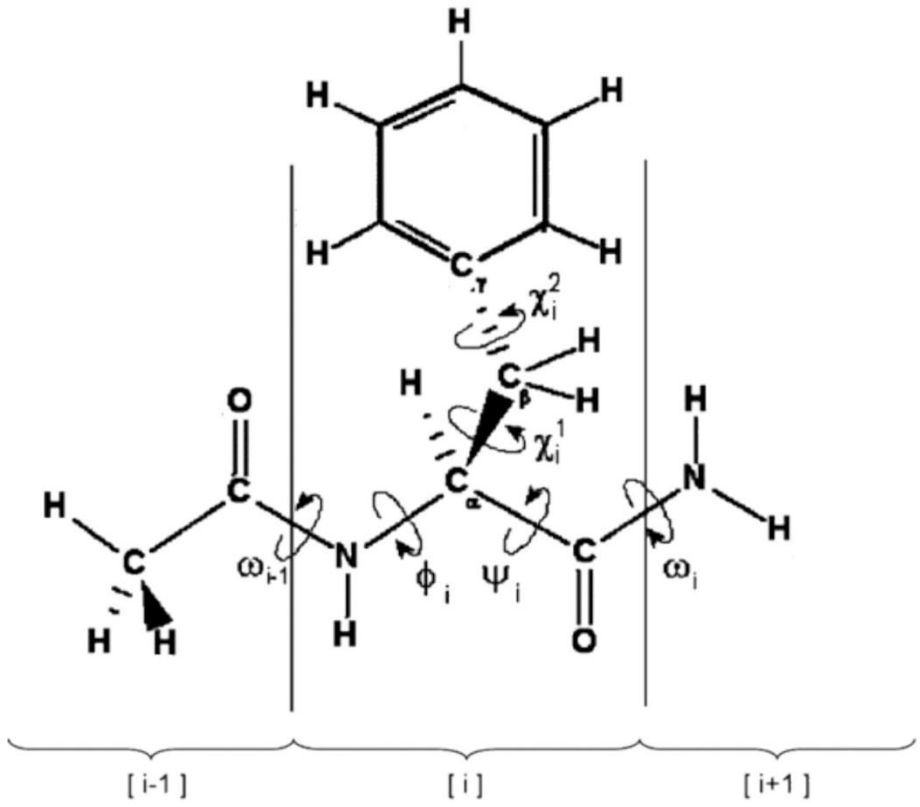

Figure S1b. Definition of the characteristic dihedral angles of the backbone of capped peptides: Example of N-Ac-Phe-NH_2 (Fa) from the N-terminus (left-most [i-1] module) through the central Phe (central [i] module) to C-terminal NH_2 protecting group (right-most [i+1] module).

S2: Nature and CC2/cc-pVDZ[3] vertical excitation energies of the five lowest excited states of Fa A, C and D at the B97-D2/TZVPP optimized geometry of the ground state.

Table S2a. Nature and vertical excitation energies of the five lowest excited states of Fa A.

Fa A	E_{vert}[a] (eV)	Nature of the state[b]	NTOs (%, occupied→virtual) [b]			
S_1	5.234	$\pi\pi^*$	**1**-55%	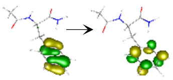	**2**-44%	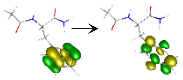
S_2	5.894	$n\pi^*_{CO(1)}$	**1**-96%	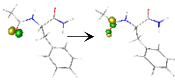		
S_3	5.985	$n\pi^*_{CO(2)}$	**1**-95%	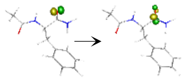		
S_4	6.456	$\pi\pi^*$	**1**-77%	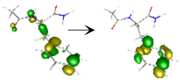	**2**-18%	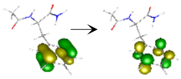
S_5	6.948	$n_{(1)}[\pi^*\pi^*_{CO(1,2)}]$ + $\pi\pi^*$	**1**-69%	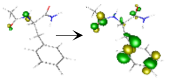	**2**-28%	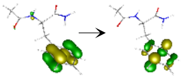

[a] The theoretical values are given with the number of significant digits obtained in the experiment. [b] In the event of several couples of NTOs, only those whose contribution to the wave function is greater than 10% are taken into account and reported. The nature of the states is determined from these contributions.

Table S2b. Nature and vertical excitation energies of the five lowest excited states of Fa C.

Fa C	E_{vert}[a] (eV)	Nature of the state[b]	NTOs (%, occupied→virtual) [b]			
S_1	5.246	$\pi\pi^*$	**1**-55%	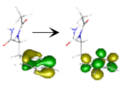	**2**-44%	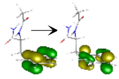
S_2	5.799	$n\pi^*_{CO(2)}$	**1**-98%	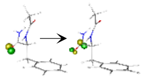		
S_3	5.966	$n\pi^*_{CO(1)}$	**1**-99%	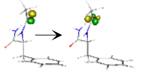		
S_4	6.556	$\pi\pi^*$	**1**-67%	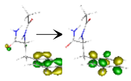	**2**-31%	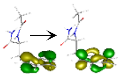
S_5	6.888	$n_{(1,2)}[\pi^*\pi^*_{CO(1)}]$	**1**-90%	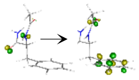		

[a] and [b] Same details as Table S2a.

Table S2c. Nature and vertical excitation energies of the five lowest excited states of Fa D.

Fa D	E_{vert}[a] (eV)	Nature of the state[b]	NTOs (%, occupied→virtual) [b]
S_1	5.260	$\pi\pi^*$	**1**-53% 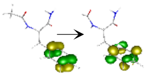 **2**-47% 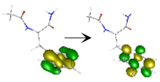
S_2	5.759	$n\pi^*_{CO(2)}$	**1**-99% 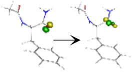
S_3	5.910	$n\pi^*_{CO(1)}$	**1**-100% 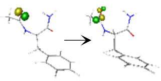
S_4	6.565	$\pi\pi^*$	**1**-69% 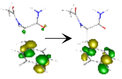 **2**-28% 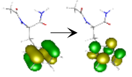
S_5	7.024	$n_{(1,2)}\pi^*_{CO(1)}$ + $[n_{(2)}\pi][\pi^*\pi^*_{CO(2)}]$	**1**-74% 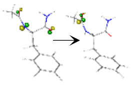 **2**-14% 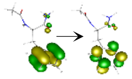

[a] and [b] Same details as Table S2a.

S3: Nature and LC-ωPBE/cc-pVDZ vertical excitation energies of the five lowest excited states of Fa A-D at the B97-D2/TZVPP optimized geometry of the ground state.

Table S3a. Nature and vertical excitation energies of the five lowest excited states of Fa A.

Fa A	E_{vert}[a] (eV)	Nature of the state[b]	NTOs (%, occupied→virtual) [b,c]	
S_1	5.482	$\pi\pi^*$	**1**- 57%	**2**- 43%
S_2	5.752	$n\pi^*_{CO(1)}$	**1**- 99%	
S_3	5.833	$n\pi^*_{CO(2)}$	**1**- 99%	
S_4	6.222	$\pi\pi^*$	**1**- 64%	**2**- 32%
S_5	7.125	$\pi\pi^*$ + $[n_{(1)}\pi][\pi^*\pi^*_{CO(1)}]$	**1**- 58% 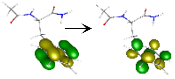	**2**- 34% 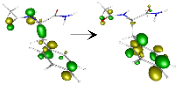

[a] The theoretical values are given with the number of significant digits obtained in the experiment. [b] In the event of several couples of NTOs, only those whose contribution to the wave function is greater than 10% are taken into account and reported. The nature of the states is determined from these contributions. [c] Only the couples of NTOs that differ from the CC2 ones are drawn.

Table S3b. Nature and vertical excitation energies of the five lowest excited states of Fa B.

Fa B	E_{vert}[a] (eV)	Nature of the state[b]	NTOs (%, occupied→virtual) [b,c]		
S_1	5.504	$\pi\pi^*$	**1**- 54%	**2**- 45%	
S_2	5.674	$n\pi^*_{CO(2)}$	**1**- 99%		
S_3	5.787	$n\pi^*_{CO(1)}$	**1**- 100%		
S_4	6.261	$\pi\pi^*$	**1**- 59%	**2**- 38%	
S_5	7.143	$\pi\pi^*$ + $[n_{(1,2)}\pi][\pi^*\ \pi^*_{CO(1)}]$ + $n\pi^*_{CO(1)}$	**1**- 48% 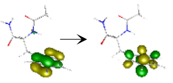	**2**- 36% 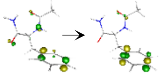	**3**- 11% 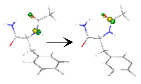

[a], [b] and [c] Same details as Table S3a.

Table S3c. Nature and vertical excitation energies of the five lowest excited states of Fa C.

Fa C	E_{vert}[a] (eV)	Nature of the state[b]	NTOs (%, occupied→virtual) [b,c]	
S_1	5.492	$\pi\pi^*$	**1**- 57%	**2**- 42%
S_2	5.645	$n\pi^*_{CO(2)}$	**1**- 99%	
S_3	5.836	$n\pi^*_{CO(1)}$	**1**- 100%	
S_4	6.262	$\pi\pi^*$	**1**- 59%	**2**- 38%
S_5	7.175	$\pi\pi^*$	**1**- 54% 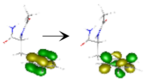	**2**- 35% 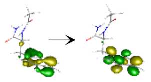

[a], [b] and [c] Same details as Table S3a.

Table S3d. Nature and vertical excitation energies of the five lowest excited states of Fa D.

Fa D	E_{vert}[a] (eV)	Nature of the state[b]	NTOs (%, occupied→virtual) [b,c]	
S_1	5.509	$\pi\pi^*$	**1**- 54%	**2**- 46%
S_2	5.622	$n\pi^*_{CO(2)}$	**1**- 99%	
S_3	5.773	$n\pi^*_{CO(1)}$	**1**- 100%	
S_4	6.271	$\pi\pi^*$	**1**- 60%	**2**- 37%
S_5	7.199	$\pi\pi^*$ + $[\pi n_{(1,2)}]\pi^*$	**1**- 55% 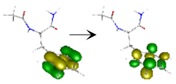	**2**- 35% 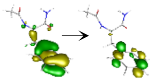

[a], [b] and [c] Same details as Table S3a.

Table S3e. Variation of the nature and vertical excitation energies of the five lowest excited states of Fa B according the value of ω (0.45-0.25 bohr⁻¹).

LC-ωPBE ω ($bohr^{-1}$)	S_1	S_2	S_3	S_4	S_5
			E(ev) Nature of the excitation and contribution to the wave function		
0.25	5.328	5.462	5.573	6.149	6.422
	$\pi\pi^*$, 100%	$n\pi^*_{CO\ (2)}$, 99%	$n\pi^*_{CO(1)}$, 100%	CT_{12},[a] 73%<br>$\pi\pi^*$, 25%	CT_{12},[b] 82%<br>$\pi\pi^*$, 16%
0.30	5.390	5.535	5.641	6.230	6.827
	$\pi\pi^*$, 100%	$n\pi^*_{CO(2)}$, 99%	$n\pi^*_{CO(1)}$, 100%	$\pi\pi^*$, 98%	CT_{12},[c] 73%<br>$\pi\pi^*$, 19%
0.35	5.449	5.605	5.712	6.257	7.038
	$\pi\pi^*$, 100%	$n\pi^*_{CO(2)}$, 99%	$n\pi^*_{CO(1)}$, 100%	$\pi\pi^*$, 97%	CT_{12},[c] 50%<br>$\pi\pi^*$, 35%
0.40	5.504	5.674	5.787	6.261	7.143
	$\pi\pi^*$, 100%	$n\pi^*_{CO(2)}$, 98%	$n\pi^*_{CO(1)}$, 99%	$\pi\pi^*$, 98%	$\pi\pi^*$, 48%<br>CT_{12},[c] 36%<br>$n\pi^*_{CO(1)}$ 11%
0.45	5.554	5.744	5.865	6.251	7.213
	$\pi\pi^*$, 100%	$n\pi^*_{CO(2)}$, 99%	$n\pi^*_{CO(1)}$, 100%	$\pi\pi^*$, 97%	$\pi\pi^*$, 87%

[a]CT_{12}: charge transfer $[n_{(1,2)}\pi]\pi^*$ excited states.
[b] CT_{12}: charge transfer $n_{(1,2)}\pi^*$ excited states.
[c]CT_{12}: charge transfer $[n_{(1,2)}\pi][\pi^*\ \pi^*_{CO(1)}]$ excited states.

Table S3f. Mean absolute error (MAE) and mean signed error (ME) (eV) between CC2 and TD-DFT level on the vertical excitation energies of the first four low-lying exited states for the conformers A and B.

ω ($bohr^{-1}$)	MAE (eV)	ME(eV)
0.25	0.28	-0.24
0.30	0.24	-0.17
0.35	0.23	-0.10
0.40	0.19	-0.07
0.45	0.17	-0.02

S4: Nature and CAM-B3LYP/cc-pVDZ vertical excitation energies of the five lowest excited states of Fa A-D at the B97-D2/TZVPP optimized geometry of the ground state.

Table S4a. Nature and vertical excitation energies of the five lowest excited states of Fa A.

Fa A	E_{vert}[a] (eV)	Nature of the state[b]	NTOs (%, occupied→virtual) [b,c]	
S_1	5.423	ππ*	**1**- 58%	**2**- 41%
S_2	5.722	nπ*$_{CO\ (1)}$	**1**- 98%	
S_3	5.807	nπ*$_{CO\ (2)}$	**1**- 96%	
S_4	6.086	ππ*	**1**- 66%	**2**- 29%
S_5	6.812	n$_{(1)}$[π*π*$_{CO\ (1,2)}$] + ππ*	**1**- 66%	**2**- 30%

[a], [b] and [c] Same details as Table S3a.

Table S4b. Nature and vertical excitation energies of the five lowest excited states of Fa B.

Fa B	E_{vert}[a] (eV)	Nature of the state[b]	NTOs (%, occupied→virtual) [b,c]	
S_1	5.450	ππ*	**1**- 55%	**2**- 44%
S_2	5.623	nπ*$_{CO\ (2)}$	**1**- 99%	
S_3	5.765	nπ*$_{CO\ (1)}$	**1**- 100%	
S_4	6.135	ππ*	**1**- 62%	**2**- 36%
S_5	6.750	n$_{(1,2)}$[π*π*$_{CO\ (1)}$] + ππ*	**1**- 82%	**2**- 14%

[a], [b] and [c] Same details as Table S3a.

Table S4c. Nature and vertical excitation energies of the five lowest excited states of Fa C.

Fa C	E_{vert}[a] (eV)	Nature of the state[b]	NTOs (%, occupied→virtual) [b,c]	
S_1	5.438	ππ*	**1**- 58%	**2**- 42%
S_2	5.608	nπ*$_{CO(2)}$	**1**- 99%	
S_3	5.813	nπ*$_{CO(1)}$	**1**- 99%	
S_4	6.139	ππ*	**1**- 61%	**2**- 37%
S_5	6.845	n$_{(1,2)}$[π*π*$_{CO(1)}$]	**1**- 93%	

[a], [b] and [c] Same details as Table S3a.

Table S4d. Nature and vertical excitation energies of the five lowest excited states of Fa D.

Fa D	E_{vert}[a] (eV)	Nature of the state[b]	NTOs (%, occupied→virtual) [b,c]			
S_1	5.457	ππ*	**1**- 54%		**2**- 45%	
S_2	5.587	nπ*$_{CO(2)}$	**1**- 99%			
S_3	5.752	nπ*$_{CO(1)}$	**1**- 100%			
S_4	6.149	ππ*	**1**- 62%		**2**- 35%	
S_5	6.969	n$_{(1,2)}$ [π*π*$_{CO(1)}$] + ππ*	**1**- 60%	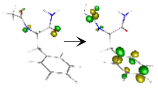	**2**- 33%	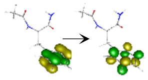

[a], [b] and [c] Same details as Table S3a.

S5: Nature and ωB97X-D/cc-pVDZ vertical excitation energies of the five lowest excited states of Fa A-D at the B97-D2/TZVPP optimized geometry of the ground state.

Table S5a. Nature and vertical excitation energies of the five lowest excited states of Fa A.

Fa A	E_{vert}[a] (eV)	Nature of the state[b]	NTOs (%, occupied→virtual) [b,c]	
S_1	5.424	$\pi\pi^*$	**1-** 58%	**2-** 42%
S_2	5.747	$n\pi^*_{CO(1)}$	**1-** 98%	
S_3	5.827	$n\pi^*_{CO(2)}$	**1-** 96%	
S_4	6.099	$\pi\pi^*$	**1-** 66%	**2-** 29%
S_5	6.836	$n_{(1)}[\pi^*\pi^*_{CO(1,2)}]$ + $\pi\pi^*$	**1-** 64%	**2-** 32%

[a], [b] and [c] Same details as Table S3a.

Table S5b. Nature and vertical excitation energies of the five lowest excited states of Fa B.

Fa B	E_{vert}[a] (eV)	Nature of the state[b]	NTOs (%, occupied→virtual) [b,c]	
S_1	5.451	$\pi\pi^*$	**1-** 55%	**2-** 45%
S_2	5.640	$n\pi^*_{CO(2)}$	**1-** 99%	
S_3	5.791	$n\pi^*_{CO(1)}$	**1-** 100%	
S_4	6.148	$\pi\pi^*$	**1-** 62%	**2-** 36%
S_5	6.785	$n_{(1,2)}[\pi^*\pi^*_{CO(1)}]$ + $\pi\pi^*$	**1-** 80%	**2-** 15%

[a], [b] and [c] Same details as Table S3a.

Table S5c. Nature and vertical excitation energies of the five lowest excited states of Fa C.

Fa C	E_{vert}[a] (eV)	Nature of the state[b]	NTOs (%, occupied→virtual) [b,c]		
S_1	5.439	$\pi\pi^*$	**1-** 58%	**2-** 42%	
S_2	5.623	$n\pi^*_{CO(2)}$	**1-** 99%		
S_3	5.842	$n\pi^*_{CO(1)}$	**1-** 99%		
S_4	6.152	$\pi\pi^*$	**1-** 60%	**2-** 37%	
S_5	6.948	$n_{(1,2)}[\pi^*\pi^*_{CO(1)}]$ + $\pi\pi^*$	**1-** 89%	**2-** 10%	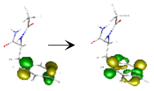

[a], [b] and [c] Same details as Table S3a.

Table S5d. Nature and vertical excitation energies of the five lowest excited states of Fa D.

Fa D	E_{vert}[a] (eV)	Nature of the state[b]	NTOs (%, occupied→virtual) [b,c]		
S_1	5.457	$\pi\pi^*$	**1**- 54%		**2**- 45%
S_2	5.598	$n\pi^*_{CO(2)}$	**1**- 99%		
S_3	5.778	$n\pi^*_{CO(1)}$	**1**- 100%		
S_4	6.161	$\pi\pi^*$	**1**- 62%		**2**- 36%
S_5	6.998	$n_{(1,2)}$ [$\pi^*\pi^*_{CO(1)}$] + [$n_{(2)}\pi$][$\pi^*\pi^*_{CO(2)}$]	**1**- 54%	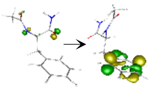	**2**- 37%

[a], [b] and [c] Same details as Table S3a.

S6: Characteristic geometrical parameters of the ground state (S_0) and the lowest excited state (S_1) of the four Fa A-D conformers and comparison of their CC2/cc-pVDZ optimized geometries[3] with those obtained at the TD-DFT level.

Table S6a: Characteristic geometrical parameters of the ground state (S_0) and the lowest $\pi\pi^*$ excited state (S_1) of the four Fa A-D conformers optimized at the CC2/cc-pVDZ level.

		Dihedral angles (°)[a]			Intramolecular distances (Å)	
		Φ	Ψ	χ^1	$d_{NH...O}$	$d_{NH...\pi}$[b]
S_1	A	-164	155	182	2.22	2.51 (2.92, 2.63)
	B	-83	52	45	1.95	2.37 (3.30, 2.35)
	C	-88	79	-50	1.99	2.52 (3.48, 2.53)
	D	-81	84	192	2.06	
S_0	A	-159	168	201	2.15	2.45 (2.54, 3.10)
	B	-82	53	44	1.96	2.37 (3.16, 2.49)
	C	-88	80	-51	1.98	2.56 (3.46, 2.70)
	D	-80	85	192	2.06	

[a] For the definition of the dihedral angles, see Figure S1b.
[b] The NH…π bond is characterized by three distances: the distance of the NH_{Phe} (A conformer) or NH_2 (B conformer and C conformer) hydrogen atom with the C_γ carbon atom of the phenylalanine residue and given in parentheses by the two distances with the two C_δ carbon atoms ($C_\delta^{C\text{-term}}$, $C_\delta^{N\text{-term}}$) of the phenylalanine residue.

Table S6b: Characteristic geometrical parameters of the ground state (S_0) and the lowest $\pi\pi^*$ excited states of the four Fa A-D conformers optimized at the CAM-B3LYP/cc-pVDZ level.

		Dihedral angles (°)[a]			Intramolecular distances (Å)	
		Φ	Ψ	χ^1	$d_{NH...O}$	$d_{NH...\pi}$[b]
S_1	A	-160	177	208	2.09	2.52 (2.49, 3.38)
	B	-82	56	45	1.92	2.42 (3.33, 2.48)
	C	-84	71	-53	1.93	2.67 (3.66, 2.76)
	D	-82	76	198	2.00	
S_0	A	-159	173	206	2.10	2.57 (2.61, 3.32)
	B	-82	57	43	1.93	2.42 (3.16, 2.65)
	C	-84	71	-54	1.94	2.74 (3.68, 2.91)
	D	-82	77	197	2.00	

[a] and [b] Same details as S6a.

Table S6c: Characteristic geometrical parameters of the ground state (S_0) and the lowest $\pi\pi^*$ excited state (S_1) of the four Fa A-D conformers optimized at the ωB97XD/cc-pVDZ level.

		Dihedral angles (°)[a]			Intramolecular distances (Å)	
		Φ	Ψ	χ^1	$d_{NH...O}$	$d_{NH...\pi}$[b]
S_1	A	-162	161	186	2.16	2.52 (2.94, 2.70)
	B	-83	54	44	1.96	2.40 (3.26, 2.46)
	C	-87	71	-51	1.97	2.60 (3.56, 2.67)
	D	-82	79	196	2.06	
S_0	A	-158	169	202	2.12	2.53 (2.62, 3.17)
	B	-82	55	42	1.96	2.40 (3.12, 2.60)
	C	-87	72	-52	1.99	2.56 (3.57, 2.83)
	D	-82	80	195	2.06	

[a], [b] and [c] Same details as Table S3a.

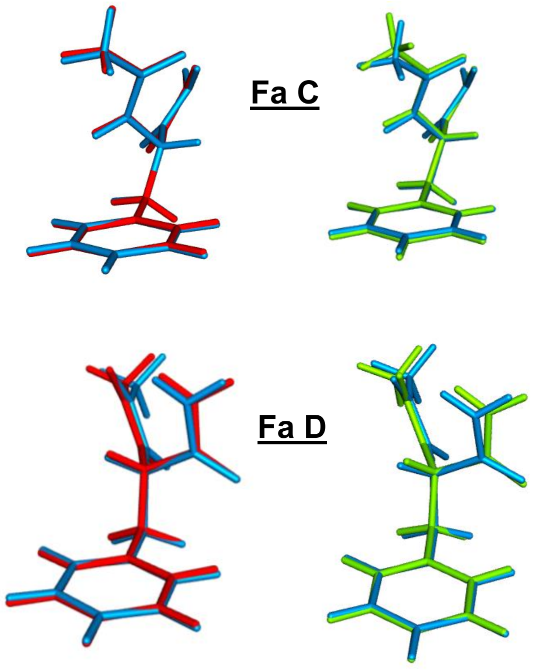

Figure S6. Comparison of the CC2/cc-pVDZ (blue) and ωB97XD/cc-pVDZ(red) as well as the CC2/cc-pVDZ (blue) and the CAM-B3LYP/cc-pVDZ (green) optimized geometries of the S_1 state for Fa C and D. For each conformer, the phenyl rings have been overlapped.

S7: ***Amide A*** **region frequencies of both the ground (S_0) and $\pi\pi^*$ excited (S_1) states of Fa A-D.**

Table S7a: CC2/cc-pVDZ *amide A* region frequencies (cm^{-1}) of both the ground (S_0) and $\pi\pi^*$ excited (S_1) states of Fa A-D,[3] together with the available experimental ones.

Conformer/State		NH_{phe}	$NH_{2\ sym.}$	$NH_{2\ anti.}$
Fa A	S_1	3594	3546	3695
	S_0	3583	3561	3713
Fa B	S_1	3545	3469	3689
	S_0	3589	3474	3690
Fa C	S_1	3553	3473	3674
	S_0	3597	3476	3675
Fa D	S_1	3618	3498	3677
	S_0	3618	3500	3677
**Experiment**				
Fa A	S_1	3434	3417	3535
	S_0	3433	3426	3541
Fa B	S_0	3438	3342	3516
Fa C	S_1	3439	3344	3514
	S_0	3463	3345	3515
Fa D	S_0	3472	3375	3516

Table S7b: CAM-B3LYP/cc-pVDZ *amide A* region frequencies (cm^{-1}) of both the ground (S_0) and $\pi\pi^*$ excited (S_1) states of Fa A-D.

Conformer/State		NH_{phe}	$NH_{2\ sym.}$	$NH_{2\ anti.}$
Fa A	S_1	3585	3557	3705
	S_0	3591	3586	3727
Fa B	S_1	3576	3463	3687
	S_0	3620	3468	3689
Fa C	S_1	3607	3468	3682
	S_0	3645	3476	3683
Fa D	S_1	3643	3495	3682
	S_0	3644	3495	3682

Table S7c: ωB97XD/cc-pVDZ *amide A* region frequencies (cm^{-1}) of both the ground (S_0) and ππ* excited (S_1) states of Fa A-D.

Conformer/State		NH_{phe}	$NH_{2\ sym.}$	$NH_{2\ anti.}$
Fa A	S_1	3626	3591	3737
	S_0	3616	3600	3745
Fa B	S_1	3609	3493	3712
	S_0	3634	3499	3715
Fa C	S_1	3609	3508	3712
	S_0	3653	3510	3710
Fa D	S_1	3661	3522	3707
	S_0	3662	3529	3708

S8: Characteristic geometrical parameters of CC2/[3] and ωB97XD/cc-pVXZ optimized geometries of both the ground (S_0) and lowest ππ* excited (S_1) states of the GFa A, FFa A_1, A_2,C and QFa A, C conformers.

Table S8a: Characteristic geometrical parameters of CC2/ and ωB97XD/cc-pVDZ optimized geometries of both the ground (S_0) and lowest ππ* excited (S_1) states of the GFa conformers.

GFa			Dihedral angles (°)[a]			Dihedral angles (°)			Intramolecular distances (Å)	
			Φ_1	Ψ_1	χ_1^1	Φ_2	Ψ_2	χ_2^1	$d_{NH...O}$[b]	$d_{NH...\pi}$[c]
CC2	S_0	A	-81	67		-84	79	-59	1.96 - 1.99	2.87 (3.86, 3.03)
		B′	56	-140		-100	14	55	2.01	2.64 (3.47, 2.67)
	S_1	A	-80	71		-86	78	-55	1.95 - 1.98	2.70 (3.54, 3.06)
		B′	56	-140		-95	10	57	2.01	2.60 (3.50, 2.56)
ωB97XD	S_0	A	-81	64		-85	71	-59	1.98 - 2.00	2.90 (3.87, 3.08)
		B′	59	-134		-101	16	54	1.99	2.68 (3.47, 2.77)
	S_1	A	-80	66		-87	69	-54	1.96 – 1.99	2.73 (3.48, 3.20)
		B′	59	-134		-98	13	55	1.99	2.66 (3.49, 2.71)

[a] For the definition of the dihedral angles, see Figure S1b. The residue 1 correspond to the first residue from the N terminal cap, the N-term.
[b] A conformer: the two distances correspond to the two C_7 hydrogen bond distances. B′ conformer, the distances is that of the C_{10} H-bond. C conformer: the two distances correspond to the two C_5 H-bond distances.
[c] The NH…π bond is characterized by three distances: the distance of NH_{phe} (A or B′ conformer) with the C_γ carbon atom of the phenylalanine residue and given in parentheses by the two distances with the two C_δ carbon atoms ($C_\delta^{to\ C\text{-}term}$, $C_\delta^{to\ N\text{-}term}$) of the phenylalanine residue.

Table S8a: Characteristic geometrical parameters of CC2 and ωB97XD/cc-pVXZ optimized geometries of both the ground (S_0) and lowest $\pi\pi^*$ excited (S_1) states of the FFa A1, A2 and C conformers.

FFa		Dihedral angles (°)[a]			Dihedral angles (°)			Intramolecular distances (Å)	
		Φ_1	Ψ_1	χ_1^1	Φ_2	Ψ_2	χ_2^1	$d_{NH...O}$[b]	$d_{NH...\pi}$[c]
S_0	A	-69	353	62	-110	14	51	2.15	[2.62, 2.54] - [2.42, 2.41]
	C	-163	151	181	-80	70	45	1.99 - 2.24	2.60 (2.94, 2.74) - 2.49 (3.41, 2.50)
S_1	A_1	-68	349	61	-103	12	52	2.10	[2.59, 2.47] - [2.43, 2.41]
	A_2	-69	352	62	-111	12	50	2.17	[2.63, 2.56] - [2.38, 2.33]
	C	-160	161	182	-73	67	41	1.96 - 2.21	2.46 (2.62, 3.15) - 2.24 (3.23, 2.17)
S_0	A	-69	351	61	-106	12	50	2.14	[2.64, 2.58] - [2.44, 2.47]
	C	-159	155	184	-82	59	47	2.03 - 2.20	2.61 (2.91, 2.85) - 2.53 (3.47, 2.60)
S_1	A_1	-69	350	60	-103	11	50	2.11	[2.61, 2.52] - [2.44, 2.49]
	A_2	-69	351	62	-107	12	48	2.16	[2.67, 2.61] - [2.39, 2.42]
	C	-161	161	185	-75	59	41	1.99 – 2.19	2.53 (2.73, 3.15) – 2.29 (3.21, 2.28)

[a] For the definition of the dihedral angles, see Figure S1b. The residue 1 correspond to the first residue from the N-term.
[b] A, A_1 and A_2 conformers: the distance corresponds to the C_{10} hydrogen bond distances. B, B_1 and B_2 conformers, the distance is that of the C_7 H-bond. C conformer: the distances correspond to the C_7 and the C_5 H-bond distances.
[c] The NH…π bond is characterized by two group of distances. A conformer: the distances of the NH_{Phe1} hydrogen atom with the C_γ and the $C_\delta^{to\ N\text{-}term}$ carbon atoms of Phe1 and those of the NH_{phe2} hydrogen atom with the C_γ and the $C_\delta^{to\ N\text{-}term}$ carbon atoms of Phe2. C conformer: the distances of the NH_{Phe2} hydrogen atom with the C_γ and the two C_δ carbon atoms ($C_\delta^{to\ C\text{-}term}$ and $C_\delta^{to\ N\text{-}term}$) of Phe1 and those of the same hydrogen atom with the C_γ and the two C_δ carbon atoms ($C_\delta^{to\ C\text{-}term}$ and $C_\delta^{to\ N\text{-}term}$) carbon atoms of Phe2.

Table S8c: Characteristic geometrical parameters of CC2/ and ωB97XD/cc-pVXZ optimized geometries of both the ground (S_0) and lowest $\pi\pi^*$ excited (S_1) states of the QFa A and C conformers.

QFa			Dihedral angles (°)[a]			Dihedral angles (°)			Intramolecular distances (Å)	
			Φ_1	Ψ_1	χ_1^1	Φ_2	Ψ_2	χ_2^1	$d_{NH...O}$[b]	$d_{NH...\pi}$[c]
CC2	S_0	A	-74	349	-63	-93	10	53	1.82 - 2.00	2.33 (3.25, 2.47)
		C	-69	343	71	-96	14	51	1.85 - 1.99	2.43 (3.19, 2.43)
	S_1	A	-65	340	-58	-106	15	47	1.83 - 2.06	2.37 (3.07, 2.35)
		C	-69	342	69	-97	14	48	1.85 - 2.00	2.39 (3.11, 2.39)
ωB97XD	S_0	A	-74	352	-63	-92	5	53	1.85 - 2.00	2.50 (3.33, 2.52)
		C	-68	345	75	-97	10	50	1.86 - 2.00	2.46 (3.24, 2.48)
	S_1	A	-74	350	-64	-90	5	53	1.85 - 1.99	2.48 (3.33, 2.46)
		C	-67	343	72	-97	11	48	1.85 - 2.00	2.42 (3.15, 2.44)

[a] For the definition of the dihedral angles, see Figure S1b. The residue 1 correspond to the first residue from the N-term.

[b] The two distances correspond to the C_7 and C_{10} H-bond distances.

[c] The NH…π bond is characterized by three distances: the distance of the NH_{Phe} with the C_γ carbon atom of the phenylalanine residue and in parentheses, the two distances with the two C_δ carbon atoms ($C_\delta^{to\ C\text{-}term}$, $C_\delta^{to\ N\text{-}term}$) of the phenylalanine residue.

S9: NTOs of the lowest ππ* excited states (S_1) of GFa A, B′, FFa A_1, A_2, C and QFa A,C at their CC2 and ωB97XD/cc-pVDZ optimized geometries.

Table S9. : NTOs of the lowest ππ* excited states (S_1) of GFa A and B at their CC2 and ωB97XD/cc-pVDZ optimized geometries.

	NTOs (%, occupied→virtual) [a]			
	CC2		ωB97XD	
GFa				
A	**1-** 58%	**2-** 42%	**1-** 61%	**2-** 39%
B	**1-** 55%	**2-** 45%	**1-** 57%	**2-** 42%
FFa				
A_1	**1-** 57%	**2-** 43%	**1-** 59%	**2-** 40%
A_2	**1-** 54%	**2-** 46%	**1-** 58%	**2-** 41%
C	**1-** 49%	**2-** 43%	**1-** 62%	**2-** 30%
QFa				
A	**1-** 55%	**2-** 45%	**1-** 56%	**2-** 42%
C	**1-** 56%	**2-** 44%	**1-** 60%	**2-** 40%

[a] In the event of several couples of NTOs, only those whose contribution to the wave function is greater than 10% are taken into account and reported.

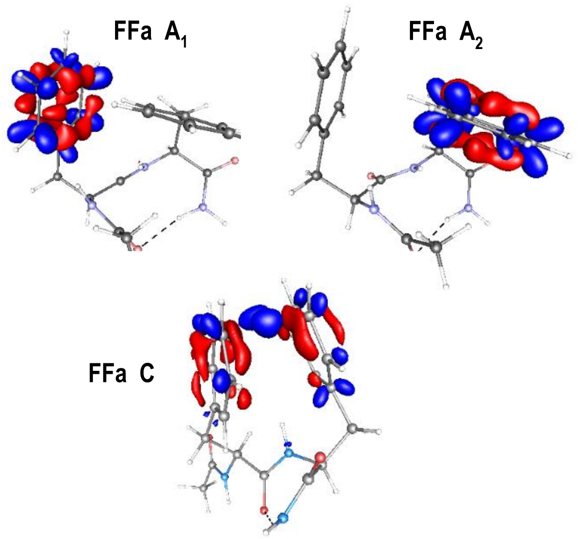

Figure S9: CC2/cc-pVDZ density difference contours (± 0.0019 au) between each of the two ππ* excited states (A_1 and A_2) and the ground state, in FFa A (top panel) and between excited and ground states in FFa C.[3] A density increase (decrease) is indicated in blue (red).

S10: A*mide A* region frequencies of both the ground (S_0) and ππ* excited (S_1) states of the GFa A, B′, FFa A_1,A_2,C and QFa A,C conformers.

Table S10a: CC2/cc-pVDZ *amide A* region frequencies (cm^{-1}) of both the ground (S_0) and ππ* excited (S_1) states of the GFa A, B′ conformers,[3] together with the IR available experimental ones (cm^{-1}).

Conformer/State		NH_{Gly}	NH_{Phe}	$NH_{2\ sym.}$	$NH_{2\ anti.}$
GFa A	S_1	3635	3434	3475	3675
	S_0	3642	3452	3480	3675
GFa B′	S_1	3626	3582	3530	3694
	S_0	3626	3603	3529	3695
**Experiment**					
GFa A	S_1	3492	3302	3358	3510
	S_0	3494	3320	3355	3519
GFa B′	S_1	3494	3423	3387	3519
	S_0	3493	3441	3385	3518

Table S10b: ωB97XD/cc-pVDZ *amide A* region frequencies (cm^{-1}) of both the ground (S_0) and ππ* excited (S_1) states of the GFa A, B′ conformers.

Conformer/State		NH_{Gly}	NH_{Phe}	$NH_{2\ sym.}$	$NH_{2\ anti.}$
GFa A	S_1	3673	3494*	3515*	3713
	S_0	3675	3504*	3512*	3712
GFa B′	S_1	3672	3641	3547	3718
	S_0	3673	3645	3546	3717

*Coupled modes. The size of the symbol indicate the strength of the coupling

Table S10c: CC2/cc-pVDZ a*mide A* region frequencies (cm^{-1}) of both the ground (S_0) and $\pi\pi^*$ excited (S_1) states of the FFa A_1, A_2, C conformers,[3] together with the IR available experimental ones (cm^{-1}).

Conformer/State		NH_{Phe1}	NH_{Phe2}	$NH_{2\ sym.}$	$NH_{2\ anti.}$
FFa A_1	S_1	3540	3587	3545	3711
FFa A_2	S_1	3576	3562	3547	3713
FFa A	S_0	3581	3596	3549	3714
FFa C	S_1	3595	3467	3458	3681
	S_0	3607	3541	3488	3692
**Experiment**					
FFa A_1	S_1	3414	3438	3390	3524
FFa A_2	S_1	3446	3414	3390	3524
FFa A	S_0	3447	3438	3391	3524
FFa C	S_0	3445	3418	3382	3518

Table S10d: ωB97XD/cc-pVDZ a*mide A* region frequencies (cm^{-1}) of both the ground (S_0) and $\pi\pi^*$ excited (S_1) states of the FFa A_1, A_2, C conformers.

Conformer/State		NH_{Phe1}	NH_{Phe2}	$NH_{2\ sym.}$	$NH_{2\ anti.}$
FFa A_1	S_1	3595	3650	3568	3729
FFa A_2	S_1	3629*	3636*	3568	3729
FFa A	S_0	3635	3657	3565	3729
FFa C	S_1	3633	3538*	3510*	3715
	S_0	3642	3598	3531	3722

*Coupled modes. The size of the symbol indicate the strength of the coupling

Table S10e: CC2/cc-pVDZ *amide A* region frequencies (cm^{-1}) of both the ground (S_0) and ππ* excited (S_1) states of the three QFa A, C conformers,[3] together with the available IR experimental ones (cm^{-1}).

Conformer/State		NH_{Gln}	NH_{Phe}	NH_2 sym./C-term	NH_2 anti./C-term	NH_2 sym./Chain	NH_2 anti./Chain
QFa A	S_1	3397	3570	3529	3705	3601	3759
	S_0	3399	3582	3521	3700	3603	3761
QFa C	S_1	3441	3560	3517	3699	3588	3740
	S_0	3450	3576	3519	3699	3587	3739
**Experiment**							
QFa A	S_0	3285	3409	3365	3519	3442	3562
QFa C	S_0	3336	3440	3367	3514	3426	3557

Table S10f: ωB97XD/cc-pVDZ *amide A* region frequencies (cm^{-1}) of both the ground (S_0) and ππ* excited (S_1) states of the three QFa A, C conformers.

Conformer/State		NH_{Gln}	NH_{Phe}	NH_2 sym./C-term	NH_2 anti./C-term	NH_2 sym./Chain	NH_2 anti./Chain
QFa A	S_1	3468	3622	3535	3718	3622	3769
	S_0	3474	3644	3537	3718	362é	3769
QFa C	S_1	3487	3610	3533	3715	3620	3767
	S_0	3496	3642	3535	3715	3618	3764

S11: Experimental vs ωB97XD/cc-pVDZ calculated harmonic *amide A* region frequencies of both the S_0 and S_1 states of the series of capped peptides and the corresponding mode-dependent linear ($\nu_{exp.} = a\nu_{theo.} + b$) scaling functions.

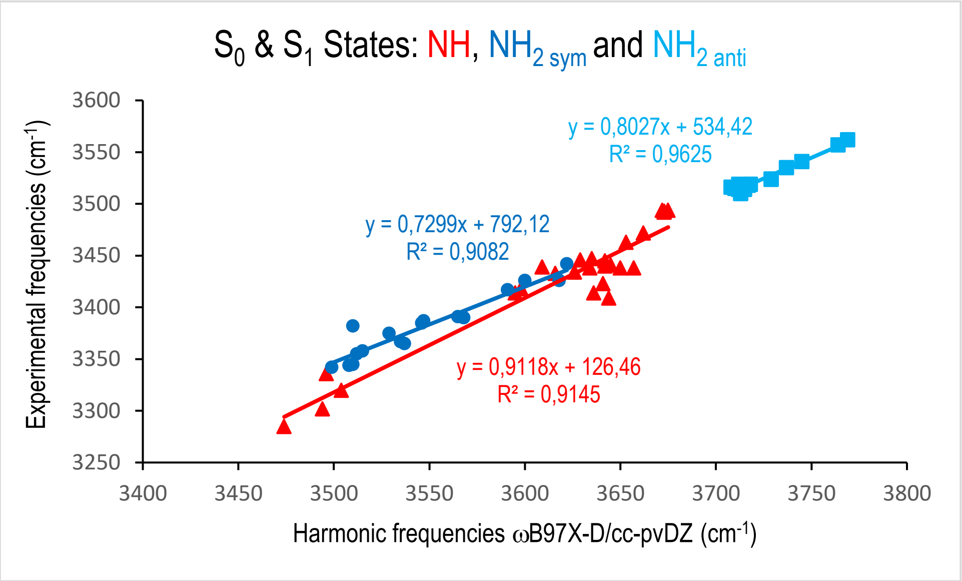

Figure S11: Experimental vs CC2/cc-pVDZ calculated harmonic *amide A* region frequencies of the S_0 and S_1 states of the series of capped peptides and the corresponding mode-dependent linear ($\nu_{exp.} = a\nu_{theo.} + b$) scaling functions.

S12: Timing of TDDFT and CC2 calculations

The timing have been determined on a dual-processor Intel Skylake 8168 fine node with 24 cores per processor for the geometry optimization (~ 25 cycles) and for the TD-DFT(ωB97X-D) frequencies calculation. The system chosen is the GFa A conformer that contains 36 atoms and 140 electrons and the basis set used is the cc-pVDZ basis set. With the CC2 method, the frequencies calculation is numerical and 216 energy and gradient calculations have been performed on 5 dual-processor Intel Skylake 8168 fine node, each calculation using 8 cores.

Table S12: Timing of the geometry optimization and frequencies calculation of GFa A (S_1) with the TD-DFT and the CC2 methods.

Timing (minutes)	TD-DFT (ωB97X-D)	CC2
$E_{optimized}(S_1)$	92	273
Frequencies(S_1)	12	480

References:

1. Chin, W.; Mons, M.; Dognon, J. P.; Piuzzi, F.; Tardivel, B.; Dimicoli, I. Competition between local conformational preferences and secondary structures in gas-phase model tripeptides as revealed by laser spectroscopy and theoretical chemistry. *Physical Chemistry Chemical Physics* **2004,** *6* (10), 2700-2709.
2. unpublished material for review only.
3. CC2 results are from M.-S. Dupuy, E. Gloaguen, B. Tardivel, M. Mons, V. Brenner, *J. Chem. Theory Comput.* **2020**, *16*, 601-611.
